# Association between Sleep Disruption and Levels of Lipids in Caucasians with Type 2 Diabetes

**DOI:** 10.1155/2013/341506

**Published:** 2013-08-29

**Authors:** Wan Aizad Wan Mahmood, Mohd Shazli Draman Yusoff, Lucy Ann Behan, Andrea Di Perna, Tommy Kyaw Tun, John McDermott, Seamus Sreenan

**Affiliations:** Department of Endocrinology and Diabetes, Royal College of Surgeons in Ireland, Connolly Hospital, Blanchardstown, Dublin 15, Ireland

## Abstract

*Aim*. To investigate the association between sleep quality and duration with lipid and glycaemic control in Caucasian subjects with type 2 diabetes. *Methods*. Sleep quality was assessed using the Pittsburgh Sleep Quality Index (PSQI) in 114 type 2 diabetes (T2DM) subjects. Comparisons were made between subjects with different sleep quality and sleep duration. Hierarchical multiple regression analyses were used to determine contributors to metabolic parameters. *Results*. Subjects with poor sleep quality (PQ; PSQI ≥ 6) had higher systolic blood pressure, glycated haemoglobin, urine albumin : creatinine ratio (UAC), total cholesterol (TC), and triglycerides (TG) (*P* < 0.05 for all) compared to those with good sleep quality (GQ; PSQI ≤ 5). Long sleep duration (LSD) subjects had higher TC and short sleep duration (SSD) subjects had higher TG compared to those with medium sleep duration. Sleep duration and PSQI score were independent predictors of TC and low-density lipoprotein cholesterol (LDL), contributing to 14.0% and 6.1% of the total variance, respectively. *Conclusions*. In this Caucasian T2DM population, PQ is associated with adverse cardiovascular risk markers, and long and short sleep disruptions have an independent negative impact on lipids. Sleep assessment should be included as part of a diabetes clinic review.

## 1. Introduction

In the latest NHANES survey [1], the estimated 10-year UKPDS risk for developing coronary heart disease among people with diabetes was 22% lower in the 2007-2008 survey compared to the 1999-2000 surveys. This decrease has been attributed to pharmacological interventions such as statins, which have become the cornerstone of treatment in patients with hyperlipidaemia. However, statins only reduce the risk of cardiovascular events by 30–40% [2] leaving a considerable residual cardiovascular risk in T2DM patients effectively treated to targets. Continuing efforts are needed to improve this residual risk by identifying any additional risk factors and improvement of the treatment modalities.

In 2008, The US National Sleep Foundation Report revealed that sleep duration on a typical workday averaged 6 hours and 40 minutes [3] and that this was significantly less than the 8.0–8.9 hours reported in a 1960 survey. This change has coincided with a dramatic increase in the prevalence of obesity, metabolic diseases, and increased cardiovascular mortality although no conclusion can be made regarding cause and effect.

Studies in sleep and mortality date back to the late 1960s when a “U” shaped relationship between short and long sleep durations and mortality was shown [4, 5]. This was further confirmed in subsequent large-scale studies [6, 7]. In the last decade, “U” shaped associations have been demonstrated between sleep duration and risk for diabetes mellitus [8], obesity [9], hypertension [10], coronary heart disease (CHD) [11], and atherosclerosis [12]. 

Specifically in T2DM subjects, a cross-sectional study in 935 women demonstrated lower high-density lipoprotein (HDL) level in normotensive subjects with short and long sleep durations compared to mid-range sleep duration [13]. A cross-sectional study in African Americans with T2DM revealed that sleep duration and sleep quality were significant predictors of glycated haemoglobin (HbA_1_c) after controlling for age, sex, BMI, insulin use, and the presence of major complications [14]. The latter study did not include data on lipid parameters, and both studies involved a homogeneous population.

Based on the evidence above, we hypothesize that sleep disruption is a potential contributor to cardiovascular disease (CVD) risk through worsening of metabolic markers and glycaemic control in male and female Caucasians with T2DM. We performed a cross-sectional study to assess associations between self-reported sleep quality, sleep duration, diabetes control, and metabolic parameters in a Caucasian diabetes cohort.

## 2. Patients and Methods

All patients with T2DM attending the diabetes clinic and diabetes day centre were eligible for the study. Inclusion criteria for the study included T2DM diagnosis based on the American Diabetes Association (ADA) criteria [15]: Caucasian in origin and able to give consent. Exclusion criteria included having other types of diabetes and inability to give consent to the study. Patients were recruited consecutively within 6-month period as they visited the centre for their diabetes review. This study was approved by the Connolly Hospital Ethics Committee. In total, 134 T2DM patients attending for annual clinic review of diabetes agreed to take part in the study. 

We performed a clinical history and recorded anthropometric data including blood pressure (BP), weight, and BMI in all patients attending our diabetes clinic. Patients were approached about the study while waiting for their turn for review. Patients who agreed to participate were brought into an interview room, where details of the study were explained, consent form was signed, and a questionnaire was filled in. At the same time, medical notes were carefully reviewed to identify any history of hypertension, hyperlipidaemia, and all current antidiabetes; lipid-lowering and antihypertensive medications were recorded. HbA_1_c, fasting glucose, fasting lipid profile, renal profile, and urine albumin: creatinine ratio (UAC) performed in our local laboratory prior to the clinic visit were also recorded.

All patients were asked to fill a questionnaire to assess their sleep quality. The Pittsburgh Sleep Quality Index (PSQI) was used. This is a validated 19-item questionnaire covering 7 components of sleep that produces a global sleep quality score that ranges from 0 to 21 [16]. A global score greater or equal to 6 distinguishes poor sleepers from good sleepers [17, 18]. As part of the PSQI questionnaire, patients were asked if they had trouble sleeping during the past month due to pain. Patients who responded positively to the question (less than once a week, once or twice weekly, or three times weekly) were identified as possibly having pain-disturbed sleep and were excluded from the analysis as pain is a known confounder [14]. Patients with GQ (PSQI score ≤ 5) were compared with patients with PQ (PSQI ≥ 6). We extracted sleep duration from the PSQI questionnaire and categorized patients into 3 groups based on their sleep duration within the last one month: short (SSD, <6 hours daily) medium (MSD, 6–8 hours daily), and long sleep durations (LSD, >8 hours daily) and comparisons between the three groups were made. 

All statistical analyses were carried out using SPSS 18.0 (SPSS Inc. Chicago, IL, USA). Continuous data are expressed as mean ± standard deviation (SD) for parametric data or median and interquartile (IQ) range for nonparametric data. Normally distributed means were compared using Student's independent *t*-test, and nonnormally distributed medians were compared using the Mann-Whitney test. Analysis of variance (ANOVA) was used to compare parametric means across 3 groups of differing sleep duration. The Kruskall-Wallis test was used when comparing nonparametric data across 3 groups with Bonferroni adjustment for significance. Categorical data were compared using the chi square test. 

For correlation and regression analyses, any nonnormally  distributed  variables  were transformed logarithmically. Pearson's correlation was used to examine the relationship between PSQI score and sleep quality status and variables of interest. Standard multiple linear regression analysis was used to examine the crude associations between sleep duration (hours) and sleep quality (PSQI score) to glucose, HbA_1_c, lipid parameters, and blood pressure (Model 1). Using hierarchical multiple regression, we repeated the analysis adjusted for age, sex, BMI, and lipid lowering treatment (Model 2). Results are presented with part correlation and *β* value, with a significance value of <0.05. We used *R* square change value to determine to what extent the variable is explained by both sleep duration and sleep quality. We squared the part correlation to determine the individual contribution of sleep duration and sleep quality to other variables. 

## 3. Results

Of the 134 patients who completed the study, 20 patients reported pain-disturbed sleep and were excluded as pain is a known confounder for disturbed sleep [14]. Therefore, 114 patients were included for analysis. All patients were Caucasians and 52 (45.6%) were females. A nightly average of nine hours of sleep within the last one month was reported in 7% of patients, 8 hours in 16.7%, 7 hours in 31.6%, 6 hours in 25.4%, 5 hours in 7.9%, 4 hours in 6.1%, and 3 hours in 5.3%, respectively. Overall, 51 patients (44.7%) had a PSQI score ≥6, indicative of poor sleep quality.

Table 1 demonstrates the comparison of baseline characteristics in patients with PQ and GQ. Patients with PQ were more likely to be females (*P* = 0.002) (Table 1). The PQ group was more likely to have higher systolic blood pressure (SBP; 147.16 ± 17.67 versus 137.30 ± 18.75 mm Hg, *P* = 0.005), HbA_1_c (50.8 (44.3–59.6) versus 43.2 (36.6–55.2) mmol/mol, *P* = 0.026), UAC (18.02 (8.19–44.27) versus 8.11 (3.12–33.1) mg/mmol, *P* = 0.03), total cholesterol (TC; 4.87 ± 0.87 versus 4.50 ± 0.87 mmol/L, *P* = 0.025), and TG (1.69 (1.26–2.44) versus 1.38 (0.97–2.07) mmol/L, *P* = 0.026). There are no significant differences between the use of insulin or oral antidiabetic medications in patients with PQ compared to GQ. 

Table 2 demonstrates the comparison of characteristics in patients with SSD, MSD, and LSD. The LSD group had higher TC compared to the MSD (5.5 ± 1.18 versus 4.55 ± 0.79 mmol/L, *P* = 0.009) while SSD group had a higher TG level compared to the MSD group (2.0 (1.44–3.02) versus 1.43 (1.0–2.08) mmol/L, *P* = 0.013, Table 2).

PSQI score correlated positively with SBP (*r* = 0.187, *P* = 0.047), TC (*r* = 0.212, *P* = 0.024), and female gender (*r* = 0.249, *P* = 0.007) and negatively with sleep duration (*r* = − 0.802, *P* < 0.001). Poor sleep quality status correlated positively with female gender (*r* = 0.309, *P* = 0.001), SBP (*r* = 0.261, *P* = 0.005), Log HbA_1_c (*r* = 0.191, *P* = 0.042), TC (*r* = 0.21, *P* = 0.025), and Log triglycerides (Log TG; *r* = 0.198, *P* = 0.035) and negatively with sleep duration (*r* = − 0.57, *P* < 0.001).

The results of multiple regression analyses examining the association between sleep measures (sleep duration and PSQI score) and glucose, HbA_1_c, lipid profiles, and blood pressure are presented in Table 3. Longer sleep duration and higher PSQI score were associated with higher TC in both the unadjusted and adjusted models contributing to 14% of the variance (*R* square change = 0.14, *P* = 0.001). Individually, in the adjusted model, sleep duration made a significant contribution of 10.4% (part correlation = 0.322) and PSQI score contributed 13.8% (part correlation = 0.372) of the variance of TC. 

In the unadjusted model, longer sleep duration was associated with higher HDL and higher PSQI score was associated with higher SBP, but these associations were no longer statistically significant in the fully adjusted model (i.e., model two). Longer sleep duration showed a trend towards correlation with higher LDL while higher PSQI score correlated with higher LDL in model one. The combination of sleep duration and PSQI score, however, was associated with higher LDL in the fully adjusted model explaining 6.1% of the variance (*R* square change = 0.061, *P* = 0.03). Individually, in the adjusted model, sleep duration contributed 5.1% (part correlation = 0.226) and PSQI score contributed 5.9% (part correlation = 0.242) of the variance of LDL cholesterol. 

In this analysis, there were no other significant associations between sleep measures and glucose, HbA_1_c, TG, level and DBP. 

## 4. Discussion

Our results have shown that sleep disruption has a potentially detrimental effect on clinical and biochemical risk markers of cardiovascular disease (CVD) in Caucasian people with T2DM. Subjects with poorer quality sleep were more likely to be females, have shorter sleep duration, higher HbA_1_c, SBP, total cholesterol, TG, and UAC. Subjects with short sleep duration had higher TG levels and those with long sleep duration had high TC levels compared to subjects who slept between 6 and 8 hours daily. However, adjusting for age, sex, BMI, and lipid-lowering treatment, we found significant associations between longer sleep duration and poorer sleep quality and TC and LDL. There was no significant difference in HbA_1_c based on sleep duration, and there was no association between PSQI score or sleep duration with HbA_1_c in multiple regression analyses. Our study is the first study to illustrate the independent association between PSQI score and sleep duration and TC and LDL cholesterol in Caucasians with diabetes.

There have only been two other studies investigating lipid parameters and sleep quality or quantity in subjects with T2DM. The Nurse's Health Study involving 935 T2DM women showed that HDL was lower in normotensive women with SSD and LSD, and frequent snoring was associated with higher TG and inversely related to HDL and adiponectin levels [13]. Whilst our study did not show any difference in HDL between groups of different sleep duration, we showed that TG was higher in SSD and nonsignificantly increased in the LSD group compared to MSD. We also showed that TC was higher in LSD compared to MSD group. The only other study in T2DM assessing sleep and metabolic risk factors was the Sleep AHEAD Study [19]. Ten sleep parameters were analyzed with six dependent metabolic variables. Apart from a weak association of sleep duration and HbA_1_c, there were no associations between other sleep parameters including sleep duration and TC, HDL, LDL, and TG. Whilst our study did not show any association between sleep measurements and HbA_1_c, we showed that sleep duration and sleep quality significantly contributes to TC and LDL cholesterol. Compared to our study, the Sleep AHEAD Study had significant methodological differences such as including subjects from a heterogeneous ethnicity, having short to normal sleep duration (mean 5.96 ± 1.21 hours/night) only, and the use of home polysomnography which has its own limitations. 

A number of other studies examining the association between sleep duration and lipid metabolism were done in nondiabetic subjects. The Hordaland Health Study, a population-based cross-sectional study in 8860 subjects without T2DM, concluded that SSD was associated with higher BMI, TC, TG, SBP, and DBP [20]. In another study involving 3995 Japanese nondiabetic subjects, women with SSD and LSD were more likely to have higher TG and lower HDL levels compared to those with MSD [21]. 

Our data have shown that compared to MSD, TC was higher in those with LSD and TG were higher in the SSD subjects. Despite the differences in patient sample and methods, our results in diabetic patients largely support the notion that long and shorter than normal sleep durations are associated with lipid abnormalities. This is significant in light of the accumulating evidence that there is a “U” shaped association between sleep duration and the risk of diabetes, obesity, hypertension, and coronary heart disease. Our results also lend further support to this emerging theory by showing independent contributions between sleep duration and sleep quality and TC and LDL.

Based on experimental and clinical data, a number of mechanisms have been suggested linking the relationship between sleep disruption and lipid metabolism. Reduced leptin, reduced insulin sensitivity, increased sympathetic nervous system activation, and increased cortisol production have all been proposed as explanations [22–24]. Further studies are needed to clearly demonstrate the pathophysiology of these findings especially in the area of adipocyte function and regulation [22]. 

Our results have demonstrated that subjects with PQ had higher HbA_1_c compared to GQ. However, there was no significant difference in HbA_1_c in groups with different sleep duration, and there were no associations between PSQI score or sleep duration and HbA_1_c in the regression analyses. This is in contrast to a previous study in an African American type 2 diabetes cohort, whereby higher perceived sleep debt (the difference between a patient's preferred sleep duration and reported sleep duration) or lower sleep quality (modified PSQI score) was associated with poorer glycaemic control after controlling for confounding factors [14]. However, differences in the method used and ethnicity of the subjects may explain the different findings compared to our study. Firstly, by subtracting sleep duration from the PSQI questionnaire, a modified PSQI score was used. Secondly, it has been suggested that African Americans take longer to fall asleep, report poorer sleep quality, less deep sleep, nap more often, and have higher prevalence of sleep-disordered breathing compared to Caucasians [25]. The findings of the current study are in agreement with a more recent study involving patients with and without diabetes, whereby habitual sleep duration assessed by wrist actigraphy was not found to be associated with markers of glucose metabolism in normal and diabetic subjects [26]. In the same study, insomnia and snoring contribute to poor sleep quality, and these factors have been shown to predict hyperglycemia and insulin resistance in type 2 diabetes. 

Our study has some limitations that need to be considered. Firstly, as this is a cross-sectional study, determination of causality cannot be established. Secondly, our sample size was small. The largest study (*n* = 935) that examined the effect of sleep on lipid metabolism in T2DM subject was the Nurses' Health Study. However, this study included only female subjects and only includes sleep duration but not sleep quality. The Sleep AHEAD Study was also larger (*n* = 305). However, as described above, the subject cohorts were different. Thirdly, we do not have any data on smoking, alcohol consumption, and physical activity/inactivity. In particular, physical activity is associated with improvement in weight and cholesterol levels [27]. Although we did not have data on self-reported activity levels, since the weight in our patients was similar in both GQ and PQ groups as well as across the three groups of different sleep duration, we do not think that this is a significant confounder. We also do not have any data on other comorbidities, depression and the use of antidepressant. In The Netherlands Study of Depression and Anxiety (NESDA) [28], lower HDL and higher TG levels were demonstrated in subjects with major depressive disorder compared to control and subjects in remission from depression. However, this association was lost after adjustment suggesting that the unfavourable lipid pattern was mainly secondary to lifestyle factors. Fourthly, we measured self-reported sleep habits using a well-validated sleep questionnaire. While some studies have reported that self-reporting of sleep habits is a reliable tool in predicting the risk of developing T2DM [29] and is considered reproducible, it has also been shown to be only moderately correlated with objectively measured sleep duration using wrist actigraphy. It has been suggested that self-reported sleep may be biased by a systematic overreporting of an average of 34 minutes for each additional hour of measured sleep [30]. Lastly, we do not have data on possible sleep-disordered breathing such as obstructive sleep apnoea (OSA), which is prevalent in patients with T2DM and is associated with poorer glycaemic control [31]. Severity of OSA has been shown to have an independent contribution to poor glycaemic control in T2DM, and studies of the effect of treating OSA on glycaemic control in T2DM have been performed. However, the results were inconsistent largely due to differences in design and duration of therapy [32]. 

Despite these limitations, to the best of our knowledge, our study is the first study to illustrate the independent association between PSQI score and sleep duration and TC and LDL cholesterol in Caucasians with diabetes. We included both genders and we have used a well-validated questionnaire to assess sleep quality and duration. 

In summary, in a Caucasian diabetic population, sleep disruption is associated with an unfavourable lipid pattern and could be an additional unrecognized risk factor for macrovascular complications in diabetes. We recommend that subjective or objective sleep assessment should be considered as part of the overall management of patients with T2DM.

## Figures and Tables

**Table 1:** Baseline characteristics based on sleep quality.

	GQ (*n* = 62)	PQ (*n* = 52)	Sig
Female	20 (31.7)	32 (62.7)	0.002
Antihypertensive	45 (71.4)	43 (84.3)	0.16
Lipid lowering	30 (47.6)	22 (43.1)	0.773
Oral antidiabetic	41 (66.1)	34 (65.4)	1.0
Insulin	14 (22.6)	8 (15.4)	0.522
Diet controlled	13 (21)	8 (15.4)	0.664
Age (years)	64.0 ± 11.32	66.0 ± 10.98	0.339
Weight (kg)	86.63 ± 21.65	83.25 ± 16.26	0.358
BMI (kg/m^2^)	30.59 ± 7.3	30.84 ± 4.36	0.835
SBP (mmHg)	137.30 ± 18.75	147.16 ± 17.67	0.005
DBP (mmHg)	76.43 ± 12.26	80.04 ± 10.35	0.097
Glucose (mmol/L)	7.2 (5.9–8.5)	7.3 (6.7–9.4)	0.169
HbA_1_c			
(%)	6.1 (5.5–7.2)	6.8 (6.2–7.6)	0.026
mmol/mol	43.2 (36.6–55.2)	50.8 (44.3–59.6)	
UAC (mg/mmol)	8.11 (3.12–33.1)	18.02 (8.19–44.27)	0.03
Total cholesterol (mmol/L)	4.50 ± 0.87	4.87 ± 0.87	0.025
TG (mmol/L)	1.38 (0.97–2.07)	1.69 (1.26–2.44)	0.026
HDL (mmol/L)	1.21 ± 0.29	1.28 ± 0.34	0.249
LDL (mmol/L)	2.51 ± 0.62	2.73 ± 0.72	0.097
Sleep duration (h)	7.25 ± 0.95	5.57 ± 1.49	<0.001

SBP: systolic blood pressure; DBP: diastolic blood pressure; BMI: body mass index; HbA_1_c: glycated haemoglobin; UAC: urinary albumin : creatinine ratio; TG: triglyceride; HDL: high-density lipoprotein; LDL: low-density lipoprotein. Genders, treatment with antihypertensive, lipid-lowering agents, oral antidiabetic medications, insulin, and diet controlled are expressed in *n* (%).

**Table 2:** Characteristics based on sleep duration.

	SSD (<6 hours, *n* = 22)	MSD (6–8 hours, *n* = 84)	LSD (>8 hours, *n* = 8)	Sig
Age (years)	64.61 ± 11.06	65.13 ± 10.82	63.17 ± 15.94	0.887
Weight (kg)	85.45 ± 18.04	85.56 ± 19.96	79.51 ± 18.66	0.702
BMI (kg/m^2^)	30.67 ± 4.6	30.91 ± 6.59	28.6 ± 4.85	0.6
SBP (mmHg)	141.14 ± 21.38	142.02 ± 18.39	140 ± 18.71	0.948
DBP (mmHg)	77.68 ± 12.05	78.37 ± 11.47	75.63 ± 12.08	0.805
Glucose (mmol/L)	7.3 (6.55–9.25)	7.2 (6.1–8.57)	8.45 (7.0–9.2)	0.304
HbA_1_c				
(%)	6.9 (6.2–7.63)	6.3 (5.6–7.18)	6.18 (5.48–8.95)	0.162
mmol/mol	51.9 (44.3–59.9)	45.4 (37.7–55.0)	44.0 (36.4–74.3)	
UAC (mg/mmol)	16.6 (8.85–38.88)	14.41 (4.1–38.27)	5.5 (2.36–9.19)	0.109
Total cholesterol (mmol/L)	4.81 ± 1.01	4.55 ± 0.79*	5.5 ± 1.18*	0.009
TG (mmol/L)	2.0 (1.44–3.02)**	1.43 (1.0–2.08)**	1.94 (1.45–2.28)	0.013
HDL (mmol/L)	1.16 ± 0.28	1.25 ± 0.32	1.35 ± 0.31	0.312
LDL (mmol/L)	2.5 ± 0.76	2.61 ± 0.64	2.86 ± 0.8	0.439

*Indicates difference between groups (*P* < 0.05). **Indicates difference between groups (*P* < 0.017 after Bonferonni adjustment).

**Table:** (a)

Outcome: Log glucose			Outcome: Log HbA_1_c		
	Model 1 (unadjusted)	Model 2 (adjusted)*		Model 1 (unadjusted)	Model 2 (adjusted)*
Sleep duration (h)			Sleep duration (h)		
*β*	0.184	0.21	*β*	0.038	−0.024
Part correlation	0.11	0.13	Part correlation	0.022	−0.014
*P*	0.25	0.19	*P*	0.813	0.886

Sleep quality (PSQI score)			Sleep quality (PSQI score)		
*β*	0.187	0.24	*β*	0.107	0.038
Part correlation	0.11	0.14	Part correlation	0.064	0.021
*P*	0.24	0.16	*P*	0.499	0.826

**Table:** (b)

Outcome: SBP			Outcome: DBP		
	Model 1 (unadjusted)	Model 2 (adjusted)*		Model 1 (unadjusted)	Model 2 (adjusted)*
Sleep duration (h)			Sleep duration (h)		
*β*	0.167	0.076	*β*	0.084	0.051
Part correlation	0.1	0.043	Part correlation	0.05	0.029
*P*	0.284	0.64	*P*	0.593	0.759

Sleep quality (PSQI score)			Sleep quality (PSQI score)		
*β*	**0.32**	0.194	*β*	0.187	0.126
Part correlation	**0.92**	0.107	Part correlation	0.112	0.07
*P*	**0.041**	0.249	*P*	0.237	0.465

**Table:** (c)

Outcome: total cholesterol			Outcome: LDL		
	Model 1 (unadjusted)	Model 2 (adjusted)*		Model 1 (unadjusted)	Model 2 (adjusted)*
Sleep duration (h)			Sleep duration (h)		
*β*	**0.506**	**0.561**	*β*	0.294	**0.393**
Part correlation	**0.302**	**0.322**	Part correlation	0.176	**0.226**
*P*	**0.001**	**0.001**	*P*	0.065	**0.016**

Sleep quality (PSQI score)			Sleep quality (PSQI score)		
*β*	**0.617**	**0.675**	*β*	**0.334**	**0.438**
Part correlation	**0.369**	**0.372**	Part correlation	**0.2**	**0.242**
*P*	**0.001**	**0.002**	*P*	**0.036**	**0.01**

**Table:** (d)

Outcome: HDL			Outcome: Log TG		
	Model 1 (unadjusted)	Model 2 (adjusted)*		Model 1 (unadjusted)	Model 2 (adjusted)*
Sleep duration (h)			Sleep duration (h)		
*β*	**0.34**	0.304	*β*	0.142	0.122
Part correlation	**0.203**	0.174	Part correlation	0.085	0.07
*P*	**0.032**	0.06	*P*	0.364	0.453

Sleep quality (PSQI score)			Sleep quality (PSQI score)		
*β*	0.285	0.214	*β*	0.285	0.279
Part correlation	0.17	0.118	Part correlation	0.17	0.154
*P*	0.072	0.201	*P*	0.07	0.101

Bold face type indicates statistically significant results. *Model 2 is adjusted for age, sex, BMI, and treatment with lipid-lowering agent.

## Abbreviations

DBP: Diastolic blood pressure
GQ: Good sleep quality
HbA_1_c: Glycated haemoglobin
HDL: HDL cholesterol
LDL: LDL cholesterol
LSD: Long sleep duration
MSD: Medium sleep duration
PSQI: Pittsburgh Sleep Quality Index
PQ: Poor sleep quality
SSD: Short sleep duration
SBP: Systolic blood pressure
TC: Total cholesterol
TG: Triglycerides
T2DM: Type 2 diabetes
UAC: Urine albumin: creatinine ratio.
